# Gamma delta (γδ) T cells in the female reproductive tract: active participants or indifferent bystanders in reproductive success?

**DOI:** 10.1093/discim/kyae004

**Published:** 2024-04-26

**Authors:** Kerrie L Foyle, Sarah A Robertson

**Affiliations:** Robinson Research Institute and School of Biomedicine, The University of Adelaide, Adelaide, SA, Australia; Robinson Research Institute and School of Biomedicine, The University of Adelaide, Adelaide, SA, Australia

**Keywords:** T cell, reproductive immunology, tolerance, uterus, pregnancy

## Abstract

The female reproductive tract accommodates and balances the unique immunological challenges of protection from sexually transmitted pathogens and tolerance of the fetus and placenta in pregnancy. Leukocytes in the female reproductive tract actively engage in extensive maternal adaptations that are imperative for embryo implantation, placental development, and fetal growth support. γδ T cells are abundant at many mucosal sites in the body, where they provide protection against pathogens and cancer, and have roles in tissue renewal and homeostasis. In this review, we summarize studies in humans and rodents showing that γδ T cells are prevalent in the female reproductive tract and fluctuate in response to hormone changes across the reproductive cycle. Emerging evidence points to a link between changes in their abundance and molecular repertoire in the uterus and pregnancy disorders including recurrent miscarriage and preterm birth. However, defining the precise functional role of female reproductive tract γδ T cells and understanding their physiological significance in reproduction and pregnancy have remained elusive. Here, we critically analyze whether reproductive tract γδ T cells could be active participants in reproductive events—or whether their principal function is immune defense, in which case they may compromise pregnancy success unless adequately regulated.

## Graphical Abstract

**Graphical Abstract:**
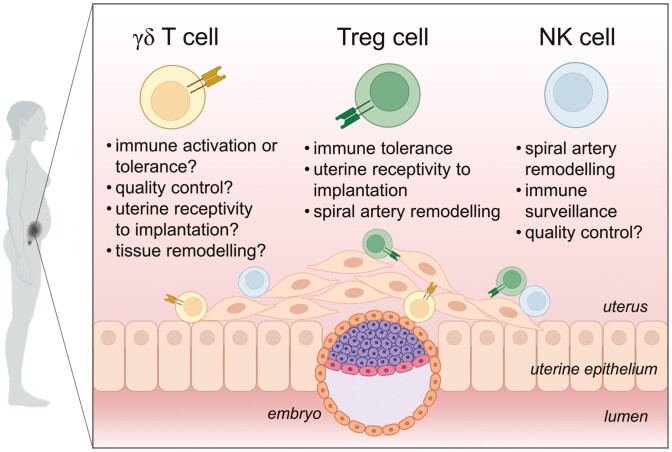

## Introduction

Female reproductive tract support of embryo implantation and development of a placenta and fetus that survive *in utero* for 9 months before birth is a remarkable feat, given the extraordinary challenge to the maternal immune system this poses. Pregnancy requires the uterus to undergo extensive tissue remodeling accompanied by local as well as systemic adaptations to maternal metabolic, vascular, and immune physiology. Failure of any of these components can cause pregnancy loss or lead to pregnancy complications resulting in poor fetal outcomes.

Events that initiate the process of reproduction in the female begin before conception and fertilization of the oocyte, with cyclical preparation of the uterus. The human menstrual cycle consists of four phases and lasts on average 26–28 days. In the luteal phase after ovulation, the endometrium (the mucosal lining of the uterus) undergoes a process of “decidualization” whereby rising progesterone causes transformation of endometrial stromal fibroblast cells (ESCs) to a specialized epithelioid decidual stromal cell (DSC) phenotype. In the absence of implantation, the decidual cells are shed during menstruation, and the cycle restarts. Mice provide a useful model to decipher the underlying mechanisms as both species have similar “hemochorial” placentation involving close contact between placental trophoblasts and maternal blood, although there are some differences in their reproductive anatomy and physiology. Notably, mice have an estrous cycle lasting only 4–5 days that, in contrast to humans, has a proliferative but not luteal phase unless mating occurs, and decidualization is dependent upon blastocyst implantation.

In a conception cycle, the blastocyst stage embryo attaches to the endometrium and trophoblasts invade into the uterine decidua [1]. In both species, implantation occurs only during the “implantation window,” a short and defined stage in the luteal phase of the cycle when the endometrium is receptive to embryo attachment and trophoblast invasion [2, 3]. A finely controlled developmental program then unfolds with successive waves of trophoblast invasion, proliferation, and differentiation to form a mature placenta that provides the developing fetus with nutrients and gas exchange, sustaining fetal growth through gestation. Development of the placenta involves remodeling of decidual blood vessels to provide adequate blood flow to the placenta, and failure of this vascular remodeling often underpins poor placental development [4].

There is a strong imperative to define how the maternal immune response adapts to tolerate the fetus and placenta in pregnancy. An immune etiology is implicated in the placental defects that underpin common reproductive conditions including recurrent implantation failure and miscarriage [5–7], as well as later onset gestational disorders such as fetal growth restriction, preeclampsia, and preterm birth [8–10]. Mouse models have provided considerable insight into the sequence of steps and the critical mediators by which the immune response supports embryo implantation and placental development, although there are limitations in the extent to which mouse data can be extrapolated to humans because of the differences in their reproductive tissues and processes.

In both species, tolerance arises in the peri-conception phase of early pregnancy as the result of interactions between maternal, paternal, and conceptus-derived signals [11, 12]. This activates a cascade of immune changes that commence before embryo implantation and persist through gestation, before delivery of the neonate at birth [13, 14]. Innate immune cells are abundant in the decidua in the luteal phase of the menstrual cycle when implantation commences, particularly macrophages [15], dendritic cells [16], and a unique population of natural killer (NK) cells with a CD56^hi^CD57^lo^ phenotype (uterine NK, or uNK cells) [17]. These cells influence placental development through immune regulation, provision of growth factors, and facilitating adaptations in the uterine vasculature to support trophoblast invasion [18].

The adaptive immune response is a central player in pregnancy tolerance [19–21] (for more information, see [14, 22]). In particular, a specific balance between regulatory T cells and effector T cells (Teffs) is required, and insufficient Treg cells are a frequent cause of impaired immune tolerance in common fertility and obstetric disorders [5–7, 9, 10]. Treg cells have a potent capacity to limit excessive inflammation and recover tissue homeostasis after insult or injury, and to suppress Teff reactions to self- or non-self antigens [23, 24]. Their anti-inflammatory, immune-regulatory, and vaso-regulatory functions collectively support embryo implantation and robust placentation to establish healthy pregnancy [23–25].

While much is known about the contributions of various leukocyte populations to tissue remodeling and immune adaptation during early pregnancy, γδ T cells remain understudied. There is emerging evidence that perturbations to γδ T cells are associated with human pregnancy complications (see later); however, the pathophysiological significance of these cells is unclear because their contribution to healthy pregnancy is not understood. In this review, we assemble emerging evidence showing that γδ T cells are abundant in the uterus, have unusual phenotypes that could afford specific functional roles, and may contribute to pregnancy tolerance in humans and mice. While there is still much to learn, we identify a range of possible activities in which these cells might participate and point to gaps in knowledge that must be addressed to better understand their physiological and pathophysiological significance.

## γδ T cells: ontogeny and tissue distribution

γδ T cells are present in all mammalian species, although they are most extensively described in humans and mice. They develop in the fetal and postnatal thymus in ontogenic waves utilizing specific T-cell receptor (TCR) recombinations [26]. γδ T cells produced in the fetal thymus have germline or oligoclonal TCRs and are pre-programmed to effector fates. These include interferon-gamma (IFNγ)-producing Tγδ1 or interleukin (IL)-17A-producing Tγδ17 cells in mice, and Type 1, Type 2, and Type 3 subsets in humans that align with classical Type 1–3 immune responses [27, 28]. Effector fate is determined by the γδTCR in mice, but not in humans [27, 29]. In both species, γδ T cells derived from the late-fetal and postnatal thymus are not pre-programmed and have polyclonal TCRs.

The Vδ chain is used to define human subsets of γδ T cells amongst which Vδ2^+^ are abundant in blood, and Vδ1^+^ and Vδ3^+^ cells are predominantly tissue resident. The Vδ2 chain usually pairs with Vγ9, and Vγ9^+^Vδ2^+^ cells have an innate lymphoid cell-like biology and recognize phosphoantigens presented on butyrophilin molecules [30, 31]. Vδ2^+^ cells with other γ chains are rare in blood and exhibit adaptive capability [30]. Vδ1^+^ cells have different Vγ chains and are adaptive cells capable of recognizing a wide range of antigens. In neonates, the Vδ1^+^ cell repertoire is highly polyclonal. By adulthood, it becomes dominated by just a few clones and unlike the public TCRs shared by the Vγ9^+^Vδ2^+^ subset, the Vδ1^+^ repertoire in the blood is very private [32–34]. Less is known about Vδ3^+^ cells, although in similarity with Vδ1^+^ cells, they exhibit a range of Vγ chains and are also capable of clonotypic expansion indicative of adaptive immunity [35, 36]. Viruses such as cytomegalovirus and other microbes appear to contribute to shaping the more focused TCR repertoire of both Vδ1^+^ and Vδ3^+^ cells that are present in adults.

Mouse γδ subsets are defined by the Vγ chain (Heilig and Tonegawa nomenclature [37]). Skin-homing Vγ5^+^ dendritic epidermal T cells are produced in the fetal thymus initially, then Vγ6^+^ cells followed by Vγ1^+^ and Vγ4^+^ subsets appear, and finally Vγ7^+^ cells are produced in the perinatal period [29]. In contrast to other subsets, production of Vγ1^+^ and Vγ4^+^ cells continues in adult mice. Specific γδ T-cell subsets colonize different tissues, including the skin, liver, lung, uterus, kidney, secondary lymphoid organs, and intestine [38, 39].

## γδ T cells as tissue sensors of stress

The unique properties of γδ T cells situate them between the innate and adaptive arms of immunity. While αβ T cells sense non-self in the context of self- major histocompatibility complex (MHC) and NK cells sense missing self, γδ T cells fill another niche by sensing in essence two classes of antigens—“safe” non-self, and stressed self [40] (Table 1). A wider diversity of antigens can be recognized by γδ T cells compared with αβ T cells, since γδ T cells are not restricted to processed peptide antigens presented on MHC molecules. γδTCR ligands include both soluble and membrane-bound molecules, MHC, small peptides, phospholipids, sulfolipids, and prenyl pyrophosphates [41]. While some of these molecules may be recognized directly, others require presentation on cell surface molecules such as butyrophilins [31]. γδ T cells also recognize group 1 CD1 molecules (CD1a, b, and c) on which lipid antigens are presented, as well as the group 2 CD1 molecule CD1d.

**Table 1:** antigen-sensing properties of uterine γδ T cells compared with αβ T cells and NK cells in the uterus

	αβ T cells	γδ T cells	NK cells
Antigen receptor	αβTCR (polyclonal)	γδTCR (polyclonal in humans, oligoclonal in mice)	n/a
Other major activating/inhibiting receptors	NKG2 receptors, NCRs (CD8^+^ T cells)	NKG2 receptors, NCRs (NKp44, NKp46, NKp30), LILRB1 (human),	NKG2 receptors, NCRs, KIRs (human), LILRB1 (human), Ly49 receptors (mice)
MHC/HLA recognition	CD4^+^ T cells: HLA-DR, -DP, -DQ/MHC class II-restricted. CD8^+^ T cells: HLA-A, -B/MHC class I-restricted	MHC-unrestricted but can recognize HLA-A, -B (MHC class I), HLA-C, -E, -G	HLA-A, -B, -C (MHC class I), HLA-E, -G
Antigens/ligands	Processed peptides on MHC. Lipids presented on CD1a, b or c. MICA and MICB (CD8^+^ T cells).	Unprocessed antigens including phospholipids, sulfolipids, prenyl pyrophosphates, in native form or presented on BTN molecules. Lipids presented on CD1a, b, c or d. MICA and MICB. Annexin A2. MR1.	Missing or altered MHC class I (predominantly HLA-C, also HLA-A, -B). Non-classical MHC (HLA-E, -G). MICA and MICB.
Immune memory	Adaptive	Adaptive or semi-adaptive (human Vδ1^+^, Vδ3^+^)	Trained (innate)
Hormone responsiveness	Estrogen and progesterone	Estrogen and progesterone	Estrogen and progesterone
Abundance in uterus	++	+ (human) +++ (mouse)	+++++
Cytotoxic subsets in uterus	CD8^+^ T cells	Human: Vδ2^+^, Vδ1^+^ (variably). Vδ3^+^ unknown. Mouse: cytotoxic potential but subsets not well defined	
Immunoregulatory subsets in uterus	Treg cells	Regulatory functions have been described but subsets not fully characterized	

TCR: T-cell receptor; HLA: human leukocyte antigen; MHC: major histocompatibility complex; KIRs: killer-cell immunoglobulin-like receptors; LILRB1: leukocyte immunoglobulin-like receptor B2; NCRs: natural cytotoxicity receptors; BTN: butyrophilin; MICA/B: MHC class I chain-related A or B; MR1: MHC Class I-related protein.

Interestingly, γδTCRs can bind more than one ligand and can provoke innate, quasi-adaptive, and adaptive γδ T-cell expansion [42]. Exactly how γδ T cells are capable of discerning and responding uniquely to different antigens via the same TCR is poorly understood, although different modalities of TCR binding or the presence of secondary signals such as cytokines can provide context for eliciting different intracellular signaling cascades that evoke specific responses [40, 41, 43]. Many of the antigens recognized by γδ T cells are cell stress-induced membrane molecules, even when adaptive clonal expansion occurs in response to infection [44].

Given the conserved roles of γδ T cells in mucosal sites, it seems highly likely that discerning “safe” non-self and stressed self from normal self will be a crucial function of γδ T cells in the female reproductive tract. If so, this raises the prospect that γδ T cells play significant roles in reproductive physiology, as the reproductive tissues are unparalleled in the degree of regular tissue remodeling they undergo to accommodate the estrous cycle and pregnancy. Healthy reproduction requires the female reproductive tract to discern and select between gametes and embryos to optimize the outcomes of reproductive investment opportunities, and the selective capabilities of γδ T cells could well contribute to this. Optimal maternal investment into good quality embryos requires active immune tolerance, a process that might reasonably include γδ T-cell detection of “safe” non-self paternally inherited allo-antigens or trophoblast lineage-specific molecules that are effectively “non-self” to the maternal immune response.

## γδ T cells in the female reproductive tract

There are considerable differences in the distribution of γδ T cells in the mouse and human reproductive tracts. This may reflect the different reproductive tract anatomy of the two species. In the human uterus, γδ T cells represent only ~2% of the leukocytes present, or 5–10% of the T cells [45, 46]. In contrast, γδ T cells are a prominent uterine leukocyte population in mice, representing ~60–80% of the T cells. In both species, they are distributed throughout the endometrium [45, 47]. Their greater prevalence in the mouse uterus might be linked to seminal fluid access to the uterine cavity in this species, which introduces substantial microbial populations that need to be cleared prior to embryo implantation. In contrast, seminal fluid delivered to the human female reproductive tract at coitus remains within the cervix, and only a small proportion of sperm and microbial flora escape the cervical mucus to reach the uterus. The human cervix and vagina also contain γδ T cells that may be involved in protection from sexually transmitted pathogens [48].

The ontology and TCR diversity of uterine γδ T cells differ between humans and mice. In fact, this considerable difference between species is a hindrance to advancing understanding of γδ T cells in the female reproductive tract. In humans, uterine γδ T cells appear to be derived from the postnatal thymus, indicating they are unlikely to have thymic effector programming [49]. In contrast, the mouse uterus is primarily populated with fetal-derived, pre-programmed Tγδ17 cells [38, 47, 50]. While γδTCR diversity in the uterus is low in both species, the diversity is relatively higher in humans where uterine γδ T cells exhibit polyclonal TCRs [34, 49]. In comparison, the mouse uterine γδTCR repertoire is largely oligoclonal [47].

In both humans and mice, more than one γδ T-cell subset populates the female reproductive tract. In non-pregnant women, both Vδ1^+^ and Vδ2^+^ cells are present in the endometrium, with Vδ1^+^ cells representing the majority subset. The polymorphism typical of Vδ1^+^ T cells explains their repertoire variance between individuals [34, 49]. Vδ1^−^Vδ2^−^ γδ T cells are also present, which are likely Vδ3^+^ cells [34]; however, they are rarely reported due to the lack of a Vδ3-specific antibody. The mouse uterus is populated mainly by Vγ6^+^ cells, as well as a proportion of Vγ4^+^ and some Vγ1^+^ cells, while Vγ5^+^ cells are rare [38, 39, 50]. Vγ6^+^ cells that dominate the γδ T-cell population in the mouse uterus are also found in the lung, tongue, and skin [38]. The Vδ1^+^ chain is used predominantly [39, 47] and most of the cells are IL-17A producing, with few expressing IFNγ [47, 50].

γδ T cells in the uterus are imprinted with a tissue-specific transcriptional program including higher expression levels of estrogen and progesterone receptors (PRs) which stimulate uterine γδ T-cell proliferation [47, 51, 52]. Estrogen upregulates mouse uterine γδ T-cell production of IL-17A [51, 53] and CXCR3 [51]. PR expression by γδ T cells positively correlates with their abundance in human peripheral blood and decidua, whereas it negatively correlates with their expression of checkpoint inhibitors T-cell immunoglobulin and ITIM domain (TIGIT) and programmed cell death 1 (PD-1), implying that progesterone signaling is favorable for γδ T-cell activity [52]. Progesterone can also enhance γδ T-cell immune-regulatory function by increasing their secretion of progesterone-induced blocking factor (PIBF), which has immunomodulatory functions in pregnancy including reducing NK cell cytotoxicity and increasing Th2-related and immunosuppressive cytokines IL-4 and IL-10 [52, 54]. PIBF also promotes decidualization and may also play a role in embryo implantation [55].

As well as direct effects of ovarian steroid hormones in uterine γδ T cells, indirect effects are likely to be mediated via hormone-regulated expression of cytokines, chemokines, and other microenvironmental regulators. For example, estrogen negatively regulates CCL20 production by uterine epithelial cells via estrogen receptor alpha, which may contribute to regulation of γδ T-cell abundance in the uterus throughout the estrus cycle, with γδ T cells peaking in diestrus when CCL20 is highest and estrogen is low [56]. Estrogen may also indirectly increase γδ T-cell production from the thymus via activation of mast cells at thymic efferent lymphatic vessels which promotes γδ T cell egress [57].

Uterine γδ T cells exhibit a tissue-resident phenotype. γδ T cells in the uterus of mice are enriched for expression of CD44, CD27, CD127, CD69, and ICOS, but have little or no CD62L or CD103 expression [45, 47]. In the human endometrium, γδ T cells express CD69 with variable CD103, both markers of tissue residency [45]. Although definitive studies are needed to identify which chemokines and receptors are used for γδ T-cell trafficking into or retention within the uterus, CXCR3 and CCR6 are implicated in mice [45, 51].

## Changes in γδ T cells in pregnancy

**Figure 1:**
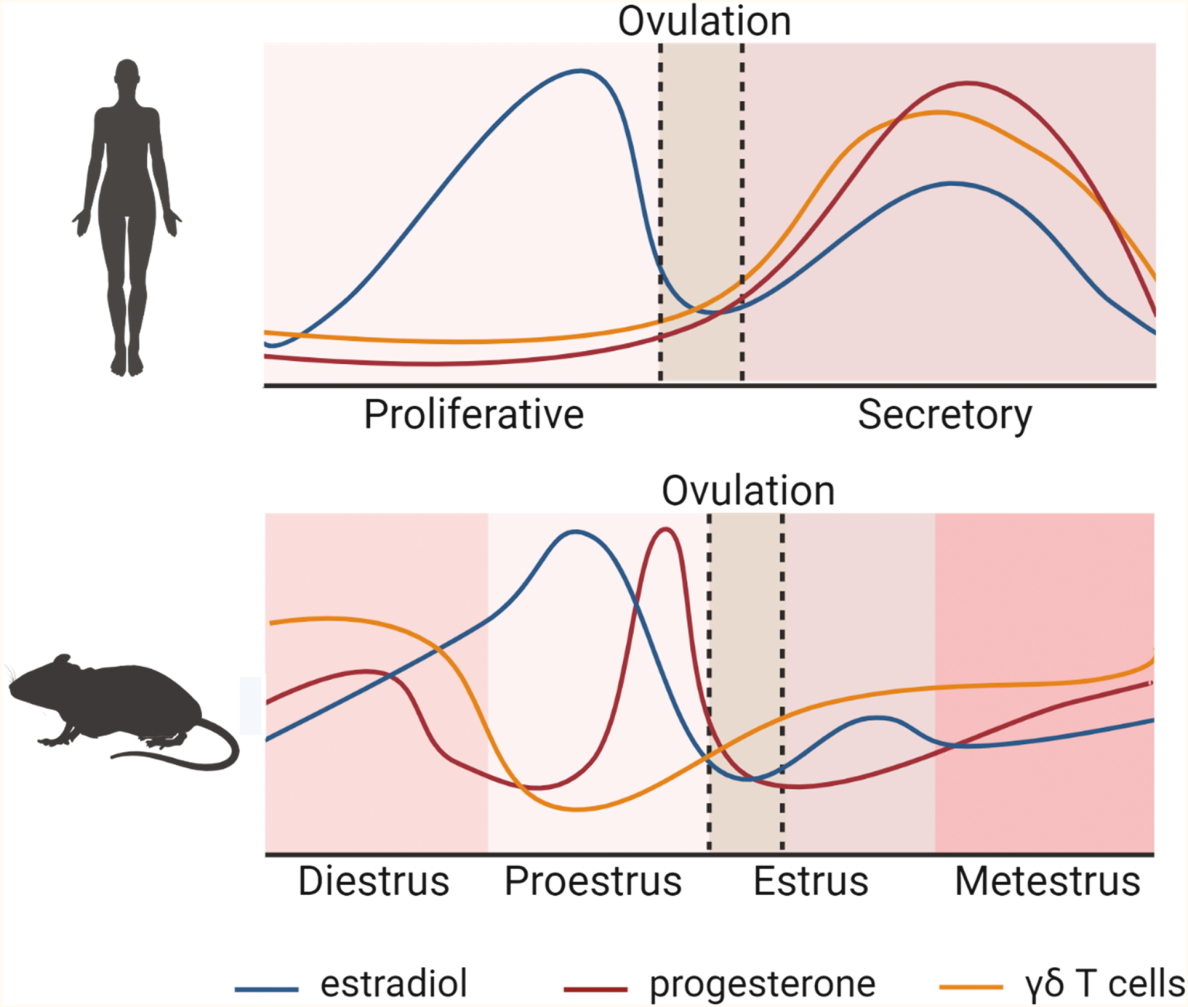
schematic showing fluctuations in uterine γδ T-cell abundance across reproductive cycles in humans and mice. In the human menstrual cycle, γδ T cells are most prevalent in the uterine endometrium when progesterone peaks in the secretory phase. Likewise in mice, abundance of γδ T cells peaks in diestrus when progesterone is high. In both species, γδ T cells remain prevalent in the uterine decidua if pregnancy occurs. The information depicted in this figure is inferred from published studies as detailed in the text. Created with BioRender.com.

γδ T-cell abundance in the uterus fluctuates across the menstrual and estrus cycles, and likely undergoes further changes in response to pregnancy (Figure 1). In the endometrium of women, γδ T cells increase from the proliferative phase to the secretory phase and remain elevated in the early decidua [46]. Similarly, γδ T-cell numbers in the uterus of mice increase at estrus and then peak as progesterone rises at diestrus, unless pregnancy occurs in which case they undergo further expansion [50]. These kinetics position uterine γδ T cells to assist in endometrial receptivity and immune adaptation for embryo implantation.

As in non-pregnant endometrium, Vδ1^+^ cells constitute the most abundant γδ T-cell subset in the early decidua in pregnant women [46, 49]. In late gestation and term decidua, the overall abundance of γδ T cells declines and the Vδ2^+^ subset predominates [46, 49]. TCR sequencing suggests that Vδ3^+^ cells increase from early to term decidua, although TRDV3 transcripts only represented a small proportion of total TRD transcripts in this study and more definitive evidence supporting this is required [49]. CD27^+^CD28^+^ naïve/memory γδ T cells increase from early to term decidua, while CD27^−^CD28^−^ effector γδ T cells that predominate in early decidua are decreased at term [34].

In pregnant mice, γδ T cells are located in both the placenta and decidua and, interestingly, the Vγ4^+^ subset is increased and Vγ6^+^ subset decreased in the placenta of allogeneic matings compared with syngeneic matings [50]. It is tempting to speculate that Vγ4^+^ cells respond to paternally delivered or inherited allo-antigens. Whether this reflects a direct interaction or the consequence of a more robust adaptive immune response in allogeneic pregnancy remains to be determined.

The ability of γδ T cells to elicit adaptive immune responses, if this occurs in pregnancy, could have implications for tolerance or rejection of the conceptus, and may affect subsequent pregnancies if a recall response occurs. Expanded clonotypes are found in human term decidua, which exhibits an overall more focused γδTCR repertoire compared with early decidua [34]. At term, there is an enrichment of oligoclonally expanded Vγ9 while Vγ2 chain abundance decreases, indicating possible suppression of early decidua γδ T cells, particularly Vγ2^+^, and infiltration or expansion of Vγ9^+^ [49]. Together, these studies suggest that γδ T cells expand in an antigen-specific manner in human pregnancy, although it is unknown whether those antigens are self- or fetal-derived and specific to pregnancy. In γδ T-cell responses to infection, TCR ligands often are aberrantly expressed molecules on the infected cell rather than specific to the microbe itself [42]. Therefore, there is a high likelihood that uterine γδ T cells in pregnancy expand in response to self-molecules associated with novel cells such as trophoblasts, or altered physiological states in uterine decidual or epithelial cells. The functional significance of antigen-specific expansion of γδ T cells in primary or next pregnancies remains to be investigated.

In mice, the uterine γδTCR has been sequenced at 2–3 weeks post-partum [47]. The diversity at this time was very low, with >99% of productive TCRs being the invariant Vγ6^+^Vδ1^+^ recombination. This could be interpreted as an indication that fetal antigen-driven γδ T-cell expansion is unlikely. However, signaling via the γδTCR may induce distinct intracellular signals for tissue status versus nominal antigens [42], meaning that γδ T cells in the uterus may nevertheless be capable of distinguishing tissue from fetal antigens via the same TCR and could mount differential responses accordingly. In the case of the invariant Vγ6^+^ uterine γδ subset, this might indicate “adaptate” immunity, a term adopted to explain their ability to expand and retain memory for antigens that are recognized by canonical TCRs present in all mice [42].

## Protection against reproductive tract pathogens and cancer

γδ T cells are ubiquitously abundant at mucosal barriers where contact with microbes is common and there is a need to distinguish between commensals that promote tissue homeostasis and function, and pathogens that threaten tissue integrity. In the female reproductive tract, γδ T cells are shown to protect against bacterial, viral, and fungal infections, although their interactions with commensal organisms require further investigation. Although γδ T-cell colonization of the female reproductive tract of mice is not dependent on microbiota [47], microbes may promote optimal γδ T-cell activity including IL-17A secretion [53]. Interestingly, the vaginal microbiome of *Tcrd*^*−/−*^ mice is unaffected by the lack of γδ T cells at least in non-pregnant mice [58]. Whether γδ T cells shape the female reproductive tract microbiome during pregnancy and if this contributes to vertical transfer of commensal microbes to offspring remains to be investigated.

In women, bacterial vaginosis has been associated with γδ T-cell dysregulation in the endocervix marked by significantly fewer Vδ1^+^ cells and increased Vδ2^+^ cells [59]. Therefore, certain species of commensal microbes may be important for the maintenance of Vδ1^+^ cells in the human female reproductive tract, and depletion of Vδ1^+^ cells associated with bacterial vaginosis may also increase the risk of HIV infection [48].

In the female reproductive tract of mice, γδ T cells protect against yeast and fungal infections such as *Candida albicans* [47] as well as viruses including herpes simplex virus (HSV)-2 [53, 60]. While IL-17A produced by both Vγ6^+^ and Vγ4^+^ cells may be important in pathogen protection, the role of other effector molecules and the contributions of Vγ6^+^ cells versus less abundant subsets remain to be determined.

Despite the normally low cytotoxic profile of endometrial γδ T cells, tumor-eradicating functions of γδ T cells appear to be not compromised in the female reproductive tract, demonstrating that their cytotoxic functions are intact when transformed cells are detected. Human endometrial tumors are enriched in Vδ1^+^ cells and their abundance correlates with patient survival indicating a likely anti-tumor role [61]. Consistent with this, endometrial cancer Vδ1^+^ cells were shown to be homogenously cytotoxic in nature. Healthy endometrium contains an amphiregulin (AREG)-expressing subset of γδ T cells that have roles in tissue homeostasis and repair in other mucosal sites [62], whereas these were absent in endometrial tumors, indicating that different subsets of γδ T cells in the uterus likely perform different functions.

In early pregnancy before placental development is complete, rapid growth of the embryo and nascent placenta causes the microenvironment to be hypoxic, restricted in nutrients, and high in oxidative stress. These conditions resemble the microenvironment of solid tumors, including a requirement for both embryo and tumor to be tolerated or evade rejection by the “host” immune system. Failure of embryonic cells to adequately adjust to this environment can result in pregnancy loss or permanent alteration to the developmental trajectory. Tγδ17 cells are metabolically programmed for increased oxidative phosphorylation (OXPHOS) so can adapt to the metabolic and physiochemical features of the tumor microenvironment, sometimes adopting a phenotype that promotes tumor growth [63]. Similarly in pregnancy, γδ T cells may suppress anti-fetal immune responses or, like uNK cells, may promote adaptations in the uterine tissue such as angiogenesis that support early pregnancy. Hypoxia in the early post-implantation environment may also reduce cytotoxicity of Tγδ17 cells [64]. Conversely, since γδ T cells are capable of utilizing their cytotoxicity against endometrial cancers, they could exhibit similar functions in identifying embryos that exhibit inadequate metabolism or growth, or signs of cellular stress. In such situations, it may be important for γδ T cells to exert cytotoxic functions to terminate the pregnancy. Such putative functions would require tightly regulated cross-talk between γδ T cells and other cell types in the uterus, including other leukocytes, endometrial or DSCs, and trophoblasts.

## Regulation of uterine γδ T-cell cytotoxicity

**Figure 2:**
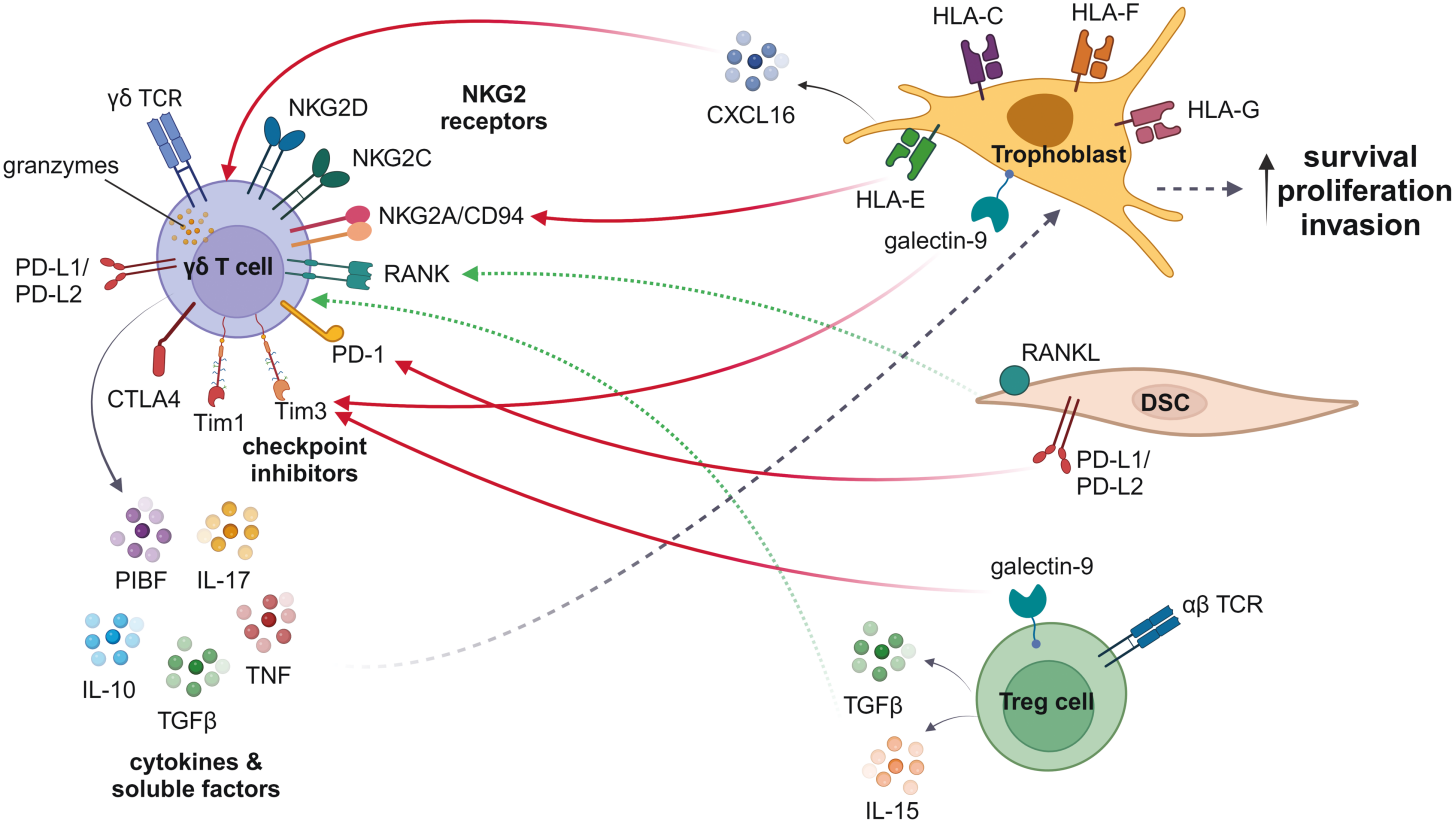
γδ T cells in healthy human pregnancy. γδ T cells are theoretically capable of recognizing HLA-C, HLA-E, HLA-F, and/or HLA-G on trophoblasts to elicit a cytotoxic response. However, interactions between γδ T cells and DSCs, trophoblasts, and other immune cells suppress γδ T-cell cytotoxicity (solid red arrows) and promote regulatory functions (dotted green arrows). Cytotoxicity against trophoblasts is regulated by γδ T-cell expression of checkpoint inhibitors, inhibitory NKG2 receptors, and low expression of granzymes and perforin. Trophoblasts downregulate γδ T-cell activity by expression of checkpoint inhibitor ligands such as galectin-9, expression of HLA-E which is recognized by inhibitory NKG2A/CD94 on γδ T cells, and production of soluble molecules such as CXCL16 to downregulate expression of granzymes. Regulatory T cells promote immunoregulatory phenotypes in γδ T cells through production of TGFβ and IL-15. Immunoregulatory γδ T cells promote trophoblast survival, growth, and invasion through production of IL-10, IL-17, TGFβ, TNF, and PIBF. The interactions depicted in this figure are inferred from published studies as detailed in the text. Created with BioRender.com.

Trophoblasts express classical MHC I (HLA-C) and non-classical MHC molecules HLA-E, HLA-F, and HLA-G that are potentially recognized by decidual and circulating γδ T cells (Figure 2). It is possible that the MHC-unrestricted manner of γδTCR antigen detection provides a layer of adaptive immune protection or quality control activity where conventional αβ T-cell-mediated immunity cannot, due to the absence of HLA-A and HLA-B expression on trophoblasts. If so, constraining potential cytotoxicity against fetal antigens in normal pregnancies must be essential, and the commitment to exert one or another functional outcome could be an important “tipping point” for pregnancy success.

Peripheral γδ T cells that express CD56 have higher expression levels of CD107a, a marker of degranulation, indicating that they may be more cytotoxic than CD56^−^ γδ T cells [65]. These CD56^+^ γδ T cells increase from about 5% to 10% of total peripheral blood γδ T cells in the second and third trimesters of pregnancy compared with non-pregnant and first-trimester pregnant women, suggesting the potential for cytotoxic responses in pregnancy that may be kept in check through PD-1 expression [65]. Moreover, early studies identified CD56^+dim^ γδ T cells in human decidua, and these cells exhibited cytoplasmic granules [66]. Decidual γδ T cells have been shown to express granzyme A, granzyme B, perforin, and FasL, indicating potential for cytotoxicity [45, 49, 67]. However, compared with blood, decidual γδ T cells express less perforin that is required for optimal granzyme activity and preferentially express granulysin, an antimicrobial pore-forming peptide like perforin that has high avidity for bacterial rather than mammalian cell membranes [49]. Although present in most mammals, the gene for granulysin is absent from the rodent genome, highlighting another difference between γδ T cells in humans versus mice.

Trophoblast expression of CXCL16 can downregulate granzyme B expression on decidual γδ T cells [68]. γδ T-cell activity at the maternal–fetal interface may be regulated by expression of NKG2 receptors, checkpoint inhibitors, and other molecules. The three main NKG2 receptors expressed by γδ T cells are the inhibitory receptor NKG2A and activating receptors NKG2C and NKG2D. While some ligands are recognized directly by NKG2 receptors, others bind in conjunction with the γδTCR. In healthy pregnancies, approximately half of early decidual γδ T cells express the activating receptor NKG2D, and this decreases slightly by term [34]. Expression of CD94/NKG2A may be important for inhibiting cytotoxicity of Vδ2^+^ cells through binding HLA-E expressed by trophoblasts [69]. In mice, less is known about expression of NKG2 receptors by uterine γδ T cells.

Checkpoint inhibitors that regulate γδ T-cell function at the maternal–fetal interface include PD-1, CTLA4, Tim-1, and Tim-3. Higher levels of Tim-1 and Tim-3 are observed on γδ T cells in the decidua compared with the spleen in mice [70, 71]. Factors produced around conception and in early pregnancy, including IL-12 and IL-18, can induce Tim-3 expression on human Vγ9^+^Vδ2^+^ T cells [72] and the Tim-3 ligand galectin-9 is expressed both in the spongiotrophoblast layer of the placenta and by decidual Treg cells [71]. Tim-3 signaling in Vγ9^+^Vδ2^+^ T cells reduces expression of granzyme B and perforin and decreases their cytotoxicity [73]. Suppression of degranulation in mouse decidual Tim-3^+^ or PD-1^+^ γδ T cells is also indicated by reduced CD107a expression [71] and mouse uterine γδ T cells are uniformly PD-1^+^ indicative of their tight regulation [47].

## Immune-regulatory effects of uterine γδ T cells

γδ T cells can exert immune-regulatory effects on other leukocytes and create an immunosuppressive environment. This is well described for several types of cancers, and some evidence points to an immune-regulatory role for γδ T cells in pregnancy. Human FOXP3^+^ regulatory γδ T cells with similar immunosuppressive properties as αβ Treg cells can be differentiated *in vitro* after stimulating Vγ9^+^Vδ2^+^ cells with TGFβ and IL-15 [74]. FoxP3^+^ γδ T cells are also observed among tumor-infiltrating γδ T cells in mice where they exert a pathological role by suppressing anti-cancer immune responses [75]. Although FoxP3^+^ γδ T cells were not found in the genital tract of naïve mice [45, 60], during pregnancy they are observed in both human and mouse decidua [76]. Interactions between human RANK^+^ γδ T cells and RANKL^+^ DSCs can induce regulatory γδ T cells that express FoxP3, TGFβ1, and increased IL-10 [76]. RANK^+^ decidual γδ T cells expressed more co-stimulatory molecules, less NKG2D, and more integrins than RANK^−^ γδ T cells, and they are less frequent in the decidua of women with recurrent miscarriage.

High levels of expression of T-cell exhaustion ligands such as PD-L1 and galectin-9 on human and mouse γδ T cells can constrain αβ T-cell responses [75]. Conversely, a potential antigen-presenting function for γδ T cells in pregnancy is also possible, since TCR-stimulated Vγ1^+^Vδ2^+^ cells upregulate the MHC class II molecule HLA-DR and co-stimulatory molecules and can present antigens to αβ T cells, inducing their proliferation similar to dendritic cells [77]. However, whether this occurs *in vivo* and in the female reproductive tract remains to be seen.

## Other potential functions of γδ T cells in reproduction

It has been reported that contact with seminal fluid deposited in the female tract at coitus leads to γδ T-cell activation [78]. A range of soluble and insoluble signaling molecules are carried in seminal fluid with the potential to activate γδ T cells, including cytokines, Toll-like receptor (TLR) ligands, heat shock proteins, and seminal extracellular vesicles that may deliver NKG2-family members or other ligands, as well as other membrane-bound molecules and phospholipids [79]. The seminal fluid microbiome is also likely involved in uterine γδ T-cell activation [80]. In mice, seminal fluid exposure induces a range of transcriptional changes in the endometrium [81], which may promote cross-talk between γδ T cells and ESCs. Other changes in the uterus occur after seminal fluid exposure, such as modified phospholipid content including altered levels of linoleic acid, arachidonic acid, saturated fatty acids, and polyunsaturated fatty acids [82]. Given that phospholipid-sensing is a feature of γδ T cells in humans and mice, its potential to activate or regulate γδ T cells in the uterus after insemination and across pregnancy should be investigated. A range of oxysterols are produced during human pregnancy, some of which are absent from non-pregnant women [83], and could also be sensed by γδ T cells [84].

IL-17A production by γδ T cells in the mouse endometrium may contribute to neutrophil recruitment after mating, trophoblast invasion, and angiogenesis [45, 85]. Despite the importance of IL-17A as a γδ T-cell effector cytokine in mice to kickstart inflammatory responses, IL-17A-secreting γδ T cells in humans are perplexingly rare. However, a small population of γδ T cells capable of producing IL-17A has been observed in the human endometrium [45]. Alternatively, human γδ T cells may use the chemokine CXCL8, which is missing from the mouse genome, as an alternative to IL-17A since it may perform some equivalent functions [42]. CXCL8 induction in the human endometrium after intercourse recruits neutrophils and enhances trophoblast invasion by increasing expression of integrins and matrix metalloproteinases [86]. It is also likely that effector molecules other than IL-17A and CXCL8 are important in modulating functions of γδ T cells in reproductive tissues. Indeed, in mice, effector molecules utilized by Tγδ17 cells are not limited to IL-17A, and a range of molecules are expressed which are important for their various functions in different settings. Human γδ T cells can also promote trophoblast growth and invasion, and protect against trophoblast apoptosis through production of IL-10 [87].

The dynamics of γδ T-cell abundance increasing in preparation for and during decidualization in both humans and mice indicates potential to contribute to this process. In mice, single-cell RNAseq predicted receptor/ligand interactions between γδ T cells and different ESC subsets during decidualization, including incoming signals to γδ T cells through PDL1, PDL2, and NKG2D and outgoing signals from γδ T cells such as IL-17, TNF, and TGFβ [88]. These predicted interactions were similar to those predicted to occur between ESCs and NK cells. While γδ T cells express many similar molecules to NK cells and they share some overlapping biological functions, γδ T cells are equipped for a broader range of sensing and therefore may regulate different processes or components of the reproductive tract [26].

## Human pregnancy disorders are associated with γδ T-cell changes

A number of studies associate changes in the abundance or balance of γδ subsets or their expression of receptors or effector molecules in the peripheral blood or at the maternal–fetal interface with pregnancy complications including recurrent miscarriage, preeclampsia, and preterm birth. In broad terms, it appears that Vδ1^+^ cells may be favorable to pregnancy whereas Vδ2^+^ cell activity may be detrimental.

In non-pregnant women experiencing recurrent miscarriage, studies measuring peripheral blood γδ T cells report inconsistent findings. One study showed no difference in γδ T-cell abundance in women with recurrent miscarriage compared with fertile women [89], whereas other studies have reported both elevated and decreased numbers [52, 90, 91]. Women with recurrent miscarriage have additionally been shown to have reduced γδ T cells in their decidua, with increased proportions of those cells expressing TIGIT or PD-1 [52]. During healthy pregnancy, peripheral blood γδ T cells increase in abundance [89], whereas pregnant women destined to miscarry or experience preterm birth had fewer γδ T cells [89, 91].

Imbalances in the subsets of γδ T cells are also associated with adverse pregnancy outcomes. Healthy pregnancy is usually associated with more Vδ1^+^ cells and less Vδ2^+^ cells. Evidence points to the possibility that Vδ2^+^ cells are functionally unchecked or increased in several pregnancy disorders. Pregnant women with a history of recurrent miscarriage as well as second- or third-trimester women at risk of preterm birth have decreased circulating Vδ1^+^ cells and increased Vδ2^+^ cells compared with healthy pregnant women [55, 92]. In the first trimester decidua, Vδ1^+^ cells outnumber Vδ2^+^ cells in healthy pregnancies while Vδ2^+^ cells predominate in recurrent miscarriage patients [46]. This was not seen in non-pregnant women experiencing recurrent miscarriage, where there was no difference in the proportion or number of γδ T cells or their localization in the endometrium [93]. Similarly in preeclampsia, no association with altered γδ T cells in the decidua has been found [94].

Dysregulation of NKG2 receptor expression in complicated pregnancies further indicates that unchecked γδ T-cell activity may be linked to these pathologies. Increased frequency of γδ T cells expressing the activating receptor NKG2D was found in women with recurrent miscarriage and was negatively associated with live birth [95]. Women at risk of preterm birth showed reduced inhibitory CD94/NKG2A expression on Vδ2^+^ cells, also indicating an increased propensity for activation [92]. Reduced NKG2A expression was found among circulating Vδ2^+^ cells in women with preeclampsia, in association with increased activating NKG2C receptor expression compared with non-pregnant and healthy pregnant women, while NKG2A/NKG2C co-expression on Vδ2^+^ cells was reduced, suggesting lower constraint of Vδ2^+^ cell activity [96].

Cytotoxicity of γδ T cells is reportedly increased in pregnancy disorders. Circulating γδ T cells more often expressed granzyme B in recurrent miscarriage patients with failed pregnancy outcomes compared with those who achieved pregnancy and fertile controls [90, 97]. Peripheral blood Vδ2^+^ cells have been shown to express more CD107a in recurrent miscarriage versus healthy pregnant women [98]. Furthermore, reduced PD-1 expression by decidual Vδ2^+^ cells in women with recurrent miscarriage may increase their cytotoxicity [99], potentially exacerbated by decreased RANK signaling and fewer RANK^+^ regulatory γδ T cells in the decidua [76].

## γδ T cells in reproductive success in mice

*Tcrd* ^*−/−*^ mice, which lack all γδ T cells, have been reported to have normal pregnancy outcomes [47]. However, for various reasons, this study does not preclude a possible physiological role for γδ T cells in pregnancy. Importantly, in *Tcrd*^*−/−-*^ mice, the absence of γδ T cells over the course of development might elicit adaptive compensatory effects. In the absence of γδ T cells, leukocytes such as Th17 cells or Type 3 innate lymphoid cells (ILC3s), which are abundant at mucosal surfaces and provide immunity against extracellular microbes, occupy their niche and recapitulate their functional roles, as demonstrated by studies in a conditional γδ T-cell knock-out mouse that circumvents this limitation [100]. Another constraint of the *Tcrd*^*−/−*^ mouse is that broadly targeting γδ T cells may hide the individual contributions of the different subsets co-existing in the uterus. As such, a complete absence of γδ T cells may affect a pregnancy differently to an imbalance in the subsets or function of γδ T cells.

In an abortion-prone model of pregnancy using CBA/J females mated with DBA/2J males, no differences were found in the abundance of γδ T cells including the different Vγ subsets, or their expression of IL-17A, IFNγ, or IL-10 compared with control CBA/J females mated to BALB/c males, suggesting that γδ T cells do not contribute to pregnancy loss [101]. Although Vγ6 mRNA expression in the uterus declined between early- (GD4.5) to mid- (GD12.5) gestation in control pregnancies but not abortion-prone pregnancies, this subset was equally abundant at the cellular level in both mating combinations. Unfortunately, that study did not investigate expression of other factors produced by γδ T cells, such as TNF, or functionally test their potential contribution to fetal resorption. A different study similarly showed no significant change in resorption rates after anti-TCRδ GL4-antibody administration [102]. However, when ultrasonic stress was used to exacerbate pregnancy loss in that study, monoclonal GL4 antibody administration had a protective effect. This suggests that γδ T cells are involved in eliciting withdrawal of maternal investment in pregnancy in the presence of adverse external factors. However, an important note is that anti-TCRδ monoclonal antibodies can be internalized so that cells persist invisibly [103], raising the question of whether complete depletion of γδ T cells was achieved in this study and whether the effects might instead be mediated by inhibiting TCR signaling. In a follow-up study, injection of anti-Vγ1.1 was able to fully recapitulate the protective effects of anti-TCRδ, implying that the Vγ1^+^ subset of uterine γδ T cells is important for inducing fetal loss in this model [104]. Also, after stress induction, there were increased TNF^+^ and fewer TGFβ2^+^ γδ T cells, which are likely pro- and anti-abortive, respectively. Significantly, anti-Vγ1.1 treatment reversed this cytokine shift.

Another study also indicates that γδ T cells may come into play in the event of a proinflammatory challenge to pregnancy. *Tcrd*^*−/−*^ mice were resistant to preeclampsia-like symptoms induced by mid–late gestation treatment with TLR agonists, suggesting that γδ T cells contribute to adverse pregnancy outcomes under inflammatory conditions [105].

## Open questions and future directions

Although interest in γδ T cells in reproductive biology is increasing, they remain an incredibly understudied cell population in the female reproductive tract. In part, this reflects the fact that much about γδ T cell sensing and biology in general is still unknown. However, significant recent advances in the field, with the availability of new methodologies and investigative approaches such as single-cell TCR sequencing to link TCR to cell phenotype and function, reasonably would shed light on the nature of female reproductive tract γδ T cells and their potential functions in reproduction. Importantly, γδ T cells exhibit greater complexity in their biology than was initially appreciated. Future investigations should therefore take into account the greater diversity of γδ T-cell phenotypes beyond their Vδ chain classification and determine whether their innate or adaptive functions, or both, may modulate pregnancy outcomes.

The evidence available thus far suggests an active role of γδ T cells in multiple facets of the reproductive process, including immunomodulation, embryotrophic support, trophoblast invasion, and perhaps decidualization. However, it appears equally important to constrain their cytotoxic activity during pregnancy, which normally is important for protection against microbes and cancer, but may be harmful to the fetus. Although mouse studies imply that γδ T cells are not critically important for pregnancy to occur, they do point to potentially important functions in fetal abortion under adverse pregnancy conditions. Unfortunately, it is not yet known if the alterations to peripheral or decidual γδ T cells in human pregnancy pathologies are causal or consequential to those conditions.

Several important questions remain to be investigated. For example, do systemic viral infections, sexually transmitted infection exposure, or specific reproductive tract microbes alter the function or balance of γδ T cells? Could this be unfavorable for reproductive success? Do the different TCR subsets of γδ T cells perform different functions in the uterus? What antigens are γδ T cells responding to during pregnancy, and why do mouse uterine γδ T cells appear not to mount an adaptive immune response to pregnancy in the same way that human γδ T cells do? Does the γδ T-cell capacity to recognize stressed or altered-self play a role in reproductive quality control? As our understanding of γδ T-cell biology advances—especially knowledge of how γδ T cells recognize and distinguish antigens and the biology behind how distinct antigens elicit different responses via the same TCR—it seems the challenge to answer these questions and understand their function in reproduction will be within reach.

## Data Availability

Not applicable.

## Abbreviations

DSCs: decidual stromal cells
ESCs: endometrial stromal fibroblast cells
IFNγ: interferon-gamma
IL: interleukin
MHC: major histocompatibility complex
OXPHOS: oxidative phosphorylation
PD-1: programmed cell death 1
PIBF: progesterone-induced blocking factor
PRs: progesterone receptors
TCR: T-cell receptor
Teffs: effector T cells
